# Improving Wait Time for Patients in a Pediatric Echocardiography Laboratory - a Quality Improvement Project

**DOI:** 10.1097/pq9.0000000000000083

**Published:** 2018-06-06

**Authors:** Anitha Parthiban, Ashley Warta, Jennifer A. Marshall, Kimberly J. Reid, Keith Mann, Girish Shirali, Tara Swanson

**Affiliations:** From the Children’s Mercy Hospital, University of Missouri Kansas City School of Medicine, 2401 Gillham Road, Kansas City, Mo.

## Abstract

**Background:**

Prolonged wait times for echocardiograms in the outpatient pediatric cardiology clinic led to patient and provider dissatisfaction at our institution. The aims of this project were to measure our baseline performance with regard to echocardiogram wait time (EWT), to implement a formal quality improvement (QI) program to improve EWT, and to measure the impact of QI on EWT.

**Methods:**

A QI team was formed comprising of cardiologists (A.P., T.S.), sonographer (A.W.), and QI mentor (J.M.). EWT was defined as time in minutes from initiation of the order in the electronic medical record to start of the echocardiogram. Goal EWT was set as ≤ 20 minutes for 90% patients. Flowcharts were created after process observation to identify sources of potential delay contributing to EWT. QI methodology such as driver diagrams were utilized to identify interventions, which were then implemented and studied as Plan-Do-Study-Act cycles.

**Results:**

Sequential interventions included early start time, huddles involving clinic and echo laboratory staff, patient tracking system, and repurposing of a clinic room for echo. EWT was tracked for 840 patients. Mean EWT was 22.5 ± 17.5 minutes at baseline and decreased to 15.3 ± 7.8 minutes postintervention (*P* < 0.001). Postintervention, 81% of the patients waited < 20 minutes for their echo, and 98% patients waited < 30 minutes, compared with baseline numbers of 62% and 76%, respectively (*P* < 0.001).

**Conclusions:**

We were able to utilize QI methodology to derive interventions and track changes, resulting in quantifiable improvement in EWT in a busy pediatric echo laboratory.

## Abstract 2

Supplemental Digital Content is available in the text.

## INTRODUCTION

At our busy tertiary pediatric care center, prolonged wait time for echocardiograms (echo) in the outpatient pediatric cardiology clinic at the main campus was a source of patient and provider dissatisfaction with anecdotal reports of patients waiting for an hour or longer. The Institute of Medicine has defined timeliness, efficiency, and patient-centered care as 3 of 6 primary aims to help improve quality of health care in the United States.^1^ Although the detrimental effect of prolonged patient wait times on the delivery of high-value health care is difficult to quantify, existing literature suggests that the impact extends beyond short-term patient satisfaction to the global perception of the quality of care received and the likelihood of continuing care with the same provider.^2–5^ As the paradigm in health care delivery shifts toward value and quality, it has become increasingly important to improve efficiency and reduce waste.

## SPECIFIC AIMS

To improve efficiency and patient and provider satisfaction in our outpatient pediatric cardiology clinic, we aimed to measure our baseline performance with regard to echo wait time (EWT) and then implement a Quality Improvement (QI) project to improve EWT. The S.M.A.R.T. (specific, measurable, achievable, realistic and timely) aim of our project was to decrease EWT for 90% of outpatient echos in the main cardiology clinic to < 20 minutes within 1 year of initiation of the project.^6^

## METHODS

### Context

We conducted this QI project in the outpatient echo laboratory at Children’s Mercy Hospital, Kansas City. This high volume pediatric laboratory (annual volume = 17,000 echos) provides echo services at 10 locations across 2 states. Over 50% of all studies are performed at the main campus, where the outpatient pediatric cardiology facility comprises 10 clinic rooms and an on-site echo laboratory with 3 imaging rooms. Echocardiograms are performed on-demand for cardiology clinic patients while echos ordered by noncardiology providers including pediatric subspecialists and community pediatricians (“echo-only’’) are performed on a scheduled basis. Outpatient throughput relies on both clinic and echo laboratory processes. Clinic patients are registered by a receptionist, moved from the waiting room by a care assistant (CA) who measures their height, weight, and vital signs; they then wait in a clinic room to meet with the provider. When an echo is required, a nurse activates the order; once it prints, it is walked to the echo laboratory and placed in a queue. The echo begins as soon as staff and space are available.

## INTERVENTIONS

**Fig. 1.**
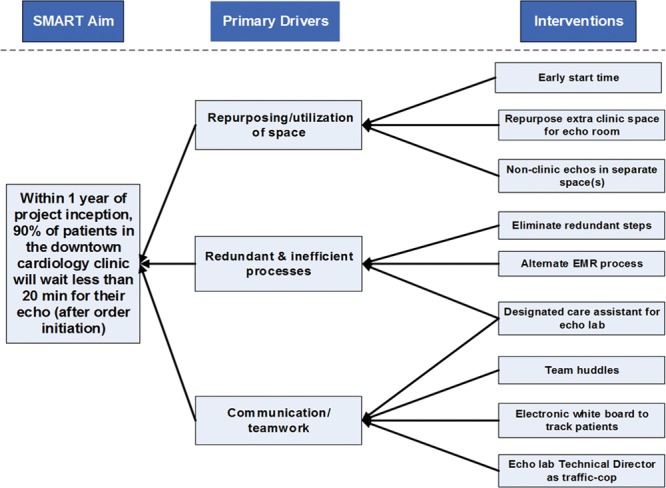
Driver diagram showing the 3 key drivers: repurposing and maximizing utilization of space, elimination of redundant steps and inefficiency, and shared ownership and improved communication among echo laboratory and clinic staff. To the far right are the interventions that affected the key drivers of the desired outcome.

A QI team was formed comprising of cardiologists (A.P., T.S.), sonographer (A.W.), and QI mentor (J.M.). This initiative was approved by the Children’s Mercy Hospital Institutional Review Board as a QI project that did not require documentation of informed consent. Key drivers of EWT were identified to focus interventions for improvement (Fig. 1). Plan-do-study-act (PDSA) cycle interventions were then sequentially implemented with follow-up data collection to assess the effect of the interventions. The interventions were based on 3 key drivers: optimized space utilization, elimination of redundant steps and inefficiency, and shared ownership and improved communication among echo laboratory and clinic staff.

### Repurposing and Maximizing Utilization of Space

Space was a significant limitation to improving patient throughput. Expansion of the clinic and echo laboratory footprint was not within the scope of this project, so alternative solutions were designed. We repurposed 1 patient room to an additional echo room to address the mismatch between the numbers of clinic and echo rooms. Nephrology and oncology echos were relocated to a repurposed imaging room in a separate (Dialysis) clinic. This redesign moved “echo only” patients off-site and allowed the 4 cardiology imaging rooms to be devoted to cardiology clinic patients. Additionally, compact imaging machines were used to perform echos in clinic rooms when the focus of the study was limited (eg, effusion check postcardiac surgery).

To maximize utilization of the 4 cardiology imaging rooms, the “Early Start” PDSA cycle addressed variable echo room utilization due to fluctuations in echo demand during clinic hours. Sonographers and space were available as early as 7 am, but there was poor utilization of the echo laboratory rooms at that time. Subsequently, the laboratory would receive clusters of orders, usually peaking between 9:30 and 10:00 am and resulting in a domino effect of causing delay. Utilization of all 3 imaging rooms by 8:00 am was established as a goal, resulting in adjustment of the “echo-only” schedule for earlier appointment times. We adjusted the cardiology clinic schedule so that it would start by 7:30 am, such that follow-up patients could have an echo performed early—even before provider evaluation.

### Elimination of Redundancy and Inefficiency

Interventions implemented to improve efficiency were (1) elimination of redundant steps; (2) improvement in workflow by alternate electronic medical record (EMR) processes; and (3) recruitment of a dedicated CA for the echo laboratory. On analysis of the baseline data and through feedback from the sonographers, it became apparent that sonographers spent a significant proportion of their time performing tasks that could potentially be delegated. For example, they escorted patients between the clinic and the echo laboratory; they also cleaned rooms and equipment between patients and walked back and forth with echo billing sheets. To decrease the amount of foot traffic for paper billing, we implemented paperless billing to allow billing via the echo reporting system itself. We altered the automated printing process in the EMR so that activated echo orders would print in the sonographers’ work area rather than in the clinic. The CA performed cleaning of rooms and equipment and escorted patients to and from their echo; as a result, sonographers were freed up to perform scans and create preliminary reports—tasks that were aligned with their expertise.

### Shared Ownership and Improved Communication

Even though the physical space of the echo laboratory is in continuity with that of the clinic, the 2 systems operated in silos. To address suboptimal communication, we initiated huddles between the clinic and echo laboratory charge staff. Information shared during huddles included the anticipated numbers of clinic patients, clinic echos ordered, echo-only appointments and staffing, and resources including number of providers in clinic and sonographer availability. Another problem was the inability to track patients effectively as they moved through the process. A dry-erase board was being used; this was inconsistently updated by the sonographers and clinic nurses. We replaced this board with an EMR-based electronic whiteboard that allowed staff to track patients; it informed providers of the patient check-in time, patient location, and total time for clinic visit. The lead clinic nurse and echo laboratory technical director were tasked with the new responsibility of continuously assessing clinic and echo laboratory traffic throughout the day. Consistent communication and coordination allowed early identification of problems and proactive implementation of solutions.

## STUDY OF THE INTERVENTIONS

We sequentially implemented interventions and monitored their effect on EWT by using Statistical Process Control charts to assess whether the interventions resulted in the desired change. We used established rules for differentiating special versus common cause variation for the charts. In addition, we compared the baseline and postintervention (after implementation of all the PDSA cycles) phases with regard to outcomes related to EWT using standard statistical tests.

## MEASURES

We defined EWT as the time interval between activation of the echo order in the EMR and the first cine acquisition in the electronic echo image storage system; this was the primary outcome measure. To target patients at highest risk for prolonged EWT, we collected data on the busiest days of the clinic. A data collection form was posted in each echo room for sonographers to capture prospectively the following: time of echo order activation, patient arrival to echo room, acquisition of first echo clip, and patient departure from echo room.

**Fig. 2.**
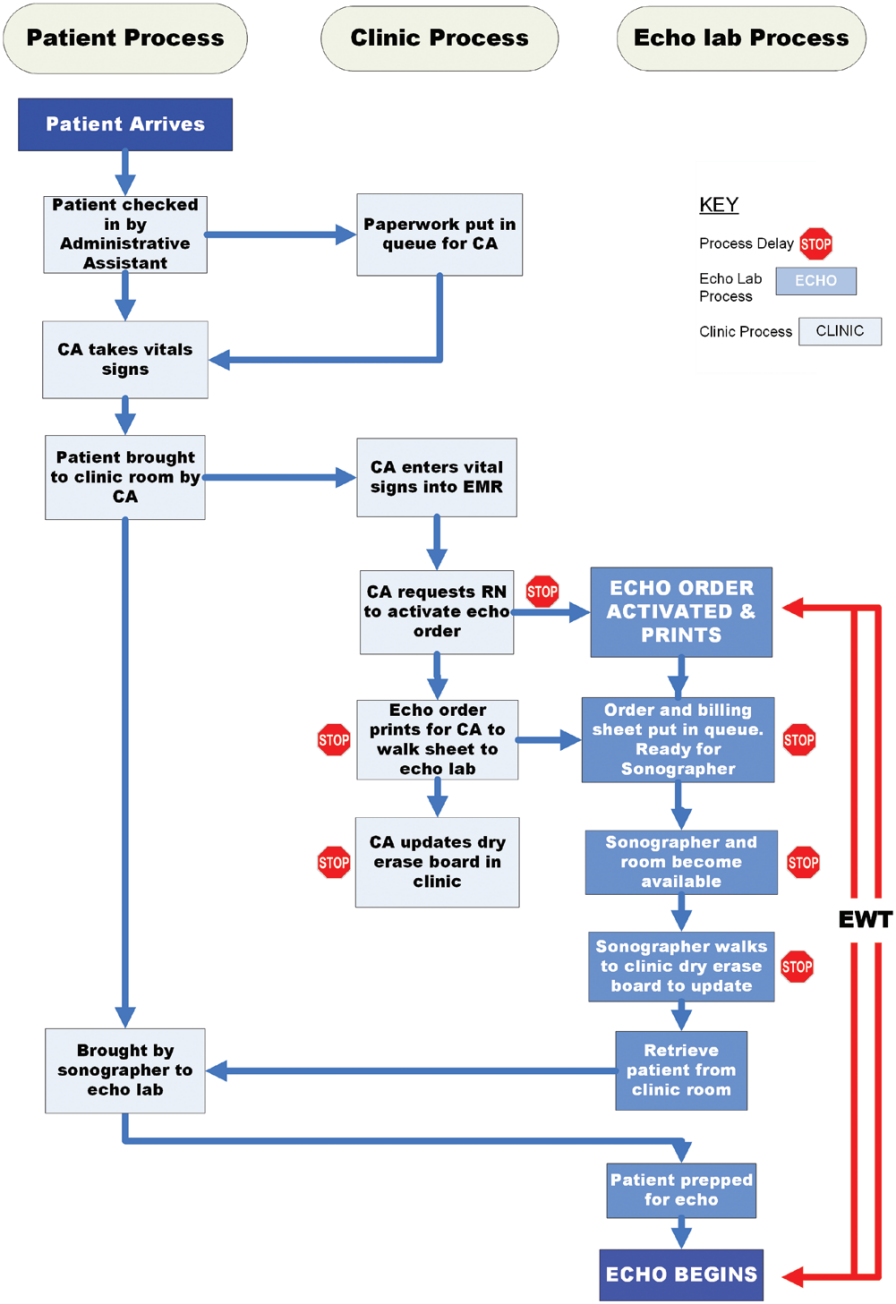
Flowchart derived from process observation. The processes seen by the patient and family are toward the left side, while the clinic and echo laboratory processes (behind the scene steps) are in the middle and right side of the figure. The red stop signs indicate the various steps where delays could occur. EWT is shown as the time taken from activation of the order to acquisition of the first image. The figure demonstrates the redundant steps such as the walking back and forth of the sonographer and CA. RN, registered nurse.

In QI methodology, balancing measures are defined as unintended negative consequences of the project. Balancing measures that we considered included the possibility that emphasizing echo laboratory efficiency may adversely affect study quality. Although we did not grade echocardiograms for quality as part of this project, we monitored the time taken for image acquisition (calculated as the time taken from the first clip to the last clip on the study). We employed direct patient observation to shadow 15 patients from the time they checked into the clinic to the start of their echo. A flow-chart detailing the steps from patient registration until echo initiation allowed for the identification of potential causes for significant delay (Fig. 2).

### Statistical Analysis

The primary outcome (EWT) was analyzed using statistical process control charts- X bar-S chart using Excel QI Macros. Special cause variation was defined as 8 data points above or below the baseline mean. Descriptive statistics are reported as means ± SD for continuous variables and frequency with percentage for categorical variables. Differences in outcomes between intervention groups were assessed using 2-sided independent t-tests for continuous variables and Chi-square or Fisher’s exact test for categorical variables, as appropriate based on cell size. Time spent in echo was highly skewed and therefore reported as median and interquartile range (IQR) and tested using Wilcoxon rank-sum test. All statistical tests were 2-sided and conducted at the alpha = 0.05 level. Statistical analysis was done using the SAS software v 9.4 (SAS Institute Inc., Cary, N.C.) and R (R Core Team, 2015: R Foundation for Statistical Computing, Vienna, Austria).

## RESULTS

Between January 27, 2014, and August 27, 2015, we tracked 840 patients for EWT. To assess if the improvement was sustained, sampling was again performed from January 22, 2016, to March 25, 2016. We divided the project into 7 phases with phase 1 being the preintervention phase, phase 7 being the postintervention phase, and the phases in between corresponding to the interventions that were implemented; these are delineated in Table 1.

**Table 1.**
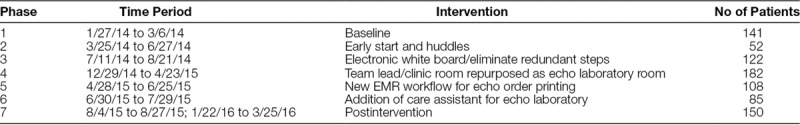
Phases of the QI Project and Number of Patients Sampled

**Fig. 3.**
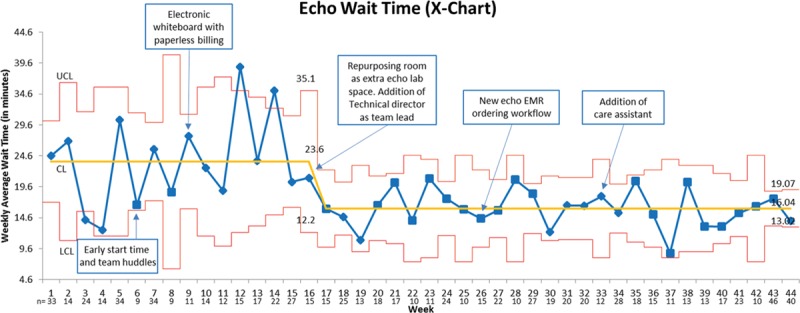
X bar chart—on the *x* axis is the timeline of the project starting from January 2014 to January 2016 and shown as weeks, while the *y* axis depicts the EWT in minutes. The timing of the various interventions is indicated by the arrows and blue boxes. A total of 840 patients were tracked across the span of the project. The yellow line or center line shows the average EWT—which was 23.6 minutes preintervention and decreased to 16 minutes at the end of the project. There was a brief period of increase in EWT around June 2014 due to severe sonographer staffing shortage. A major downward shift in the mean occurred with the third intervention, and this was sustained with subsequent interventions. The red lines are the upper and lower control lines and show a wide range in the EWT at baseline with wait times > 50 minutes; the limits became much tighter postintervention indicating that our process is more predictable and less variable. The number n on the *x* axis indicates the number of patients sampled that week.

**Fig. 4.**
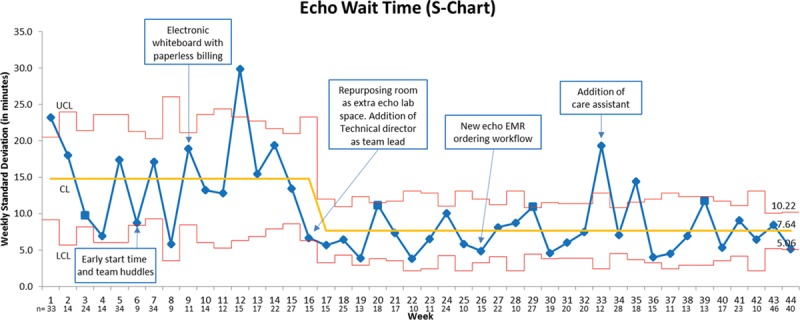
S chart- on the *y* axis is depicted the weekly SD of EWT, while the timeline of the project is shown on the *x* axis in weeks. The points represent the SD of EWT for each week sampled, whereas the solid yellow line represents the mean of the SDs. The number n on the *x* axis indicates the number of patients sampled that week. There is a decrease in the SD from 15 at baseline to 7.64 at the end of the project. A QI shift occurred after the third intervention. There is a narrowing of the overall confidence limits; however, there is still variation from patient to patient that is not yet predictable within the control limits.

Figure 3 shows the X-bar chart of the average weekly EWT; the running weekly mean EWT (solid yellow line) decreased from 23.6 minutes at baseline to 16 minutes after the implementation of the interventions by August 2015, and the effect was sustained when sampled between January and March 2016. The narrower control lines (red lines) indicate a more predictable process postintervention. A major downward shift in the weekly running mean occurred in late December 2014 with the repurposing of a clinic room as an additional echo laboratory, which also coincided with the functioning of the lead sonographer as echo laboratory traffic manager and effectively allocating resources. Figure 4 shows the S-chart of the weekly EWT; each point represents the SD for EWT for the week, and the center line (solid yellow line) represents the mean of the SDs. Similar to the X-bar chart, a downward shift is seen after implementation of the third PDSA cycle. There is a narrowing of the overall confidence limits; however, there is still variation from patient to patient that is not yet predictable within the control limits.

Table 2 depicts the characteristics of EWT at baseline and postintervention. Compared with baseline, the postintervention mean and maximal wait times decreased significantly (22.5 ± 17.5 minutes versus 15.3 ± 7.8 minutes, *P* < 0.0001, and 83 minutes versus 47 minutes, *P* < 0.001, respectively). Postintervention, there was a significant increase in patients waiting < 20 minutes (81% versus 62%, *P* < 0.0001) and < 30 minutes (97% versus 76%, *P* < 0.0001), respectively. The median time spent by the patient in echo (being actively scanned) increased postintervention compared with baseline [25.5 (IQR, 20.0–37.0) minutes versus 22.0 (IQR, 16.0–28.0) minutes, *P* < 0.0001].

**Table 2.**
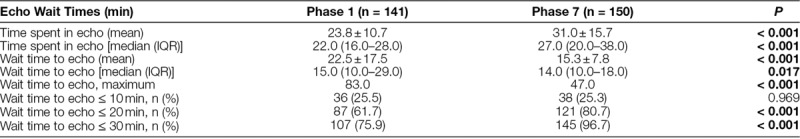
Wait Times at the End of the Project in Comparison to Baseline

## DISCUSSION

In this project, we demonstrate that process observation can be used to drive interventions that resulted in a significant and sustained improvement in wait times for echocardiograms in a busy outpatient echo laboratory setting. To our knowledge, this is the first report of a QI project to improve echo wait times in the outpatient cardiology setting.

### Choice and Design of this Project

We recognized echo wait time as a significant problem that was affecting satisfaction for both our patients and our providers. To have a specific, measurable, and well-defined aim, we defined EWT as the time from activation of the order in the EMR until the first echo clip was recorded, understanding that this also included the time taken to walk the patient to the echo laboratory and prepare them for their echo. Limiting the definition of EWT in this manner meant that EWT could be tracked retrospectively if the sonographers did not record times on their time sheet. To ensure a manageable burden of data collection, we sampled only those days that were busy for ~2 mornings a week. This strategy suggests that overall performance may be better than what was measured in this project; nevertheless, we were able to study better the days that were most adversely affected and gain insight into the nature and causes of the delays and also ensure that data collection was complete and accurate.

### Lessons Learned

We realized that process observation is crucial to identify areas in need of improvement. Along with initial discussions with clinic and echo laboratory personnel, process observation identified several time-consuming steps and processes that could easily be eliminated. A prioritization matrix can help select interventions and the sequence in which to implement them (**Supplementary figure 1, available as** Supplemental Digital Content at http://links.lww.com/PQ9/A29). The scale of the desirable effect of the intervention has to be balanced with the ease of implementation—the ideal intervention being high impact and requiring low to medium effort. Interventions such as paperless processes, elimination of back and forth walking with billing sheets and orders, and alteration of EMR workflow to print orders in the sonographer workspace were easy to implement and represented “low hanging fruit.” We also considered changing our echo laboratory flow from as-needed to scheduled echos for clinic patients. However, this was determined to be too drastic a change with potential undesired effects on patient experience. Similarly, implementing a system-wide change of allowing CA’s to have EMR privileges to activate orders was considered impractical and was therefore not pursued. Recruiting a CA dedicated to the echo laboratory required buy-in from the leadership due to the financial burden posed, but was pursued because it projected to have a significant impact on workflow. It was important to achieve buy-in from key stakeholders (sonographers, clinic nurses, and physicians, Heart Center leadership); we appointed champions for the project in each area and held monthly meetings to update all involved.

Out-of-the-box thinking helped us work around problems such as lack of space. Outsourcing echo-only studies to the Dialysis clinic improved patient flow for our renal patients, who previously had to walk from that clinic to the echo laboratory. Since then, our group has adopted this model of care for other specialty clinics such as the muscular dystrophy clinic and the cardio-oncology clinic. The use of a compact machine in clinic rooms to do quick follow-up echocardiograms allowed us to achieve additional efficiency. The designation of our lead sonographer as a “traffic cop” allowed for the continual assessment and redistribution of the sonographer pool between inpatient and outpatient areas to meet clinical needs.

QI projects require that the team identify and track balancing measures. There was a significant increase in the median time taken for completion of an echo from pre- to postintervention; this could be considered as indirect evidence that quality was not compromised in the interest of speed. Changes in the responsibilities of echo and clinic staff could also adversely affect job satisfaction, and optimization of clinic processes for echo throughput could adversely affect other clinical areas such as exercise or ECG laboratory workflow. Although we did not track these parameters specifically, there were no specific instances of provider/staff dissatisfaction.

As might be expected in any real-life project, we faced hurdles; a severe sonographer staffing shortage required a temporary halt to the study between August and December 2014. Not all providers were eager to embrace changes; regular and structured updates helped in this regard. Overall, this project shifted the culture among the clinic and echo laboratory personnel from a sense of helplessness to that of awareness and responsiveness with regard to wait time for patients.

Ensuring high quality in echocardiography is recognized as an important goal of individual echo laboratories; to that end, guidelines have been published by several organizations including the American Society of Echocardiography, American College of Cardiology, Inter-societal Accreditation Commission and the European Society of Echocardiography.^7–9^ Most of the published QI work in echocardiography has focused on improving interobserver and interacquisition variability in quantitative data or compliance with established guidelines.^10–14^ We have previously shown that implementation of a QI program results in significant improvement in image quality and completeness of echocardiograms in postoperative congenital heart disease patients.^15^ In this QI study, we focused on improving timeliness, which is less studied, but integral to patient care.

### Limitations

The findings of this QI project may not be generalizable across echo labs of varying structure and volume; nevertheless, we have outlined the methodology that we have used and the rationale for implementing specific interventions, which could be of use to others designing such a project. This project only tracked the EWT before commencement of the echo, we did not track any wait time that patients may have experienced while waiting for the finalized report after the echo was completed. We did not measure patient satisfaction directly because we did not have a system where this could be measured independent of the total clinic experience; therefore, it is not known if improving EWT had the intended effect of improving patient satisfaction. We also did not conduct a formal survey of the providers to assess satisfaction with the new state of patient throughput; we, however, received positive feedback informally from presentations at faculty meetings.

## CONCLUSIONS

We were able to significantly decrease echocardiography wait times in our ambulatory clinic by using QI methods. In an era when physicians are required to participate in QI, it is important for individual health care teams to devise and implement QI processes that are meaningful to their system.

## DISCLOSURE

The authors have no financial interest to declare in relation to the content of this article.

## Supplementary Material
