# Supplementary figures and images for: Improving Wait Time for Patients in a Pediatric Echocardiography Laboratory - a Quality Improvement Project
Source: Pediatr Qual Saf. 2018 Jun 6;3(3):e083. doi: 10.1097/pq9.0000000000000083 (PMC6132813; doi:10.1097/pq9.0000000000000083)

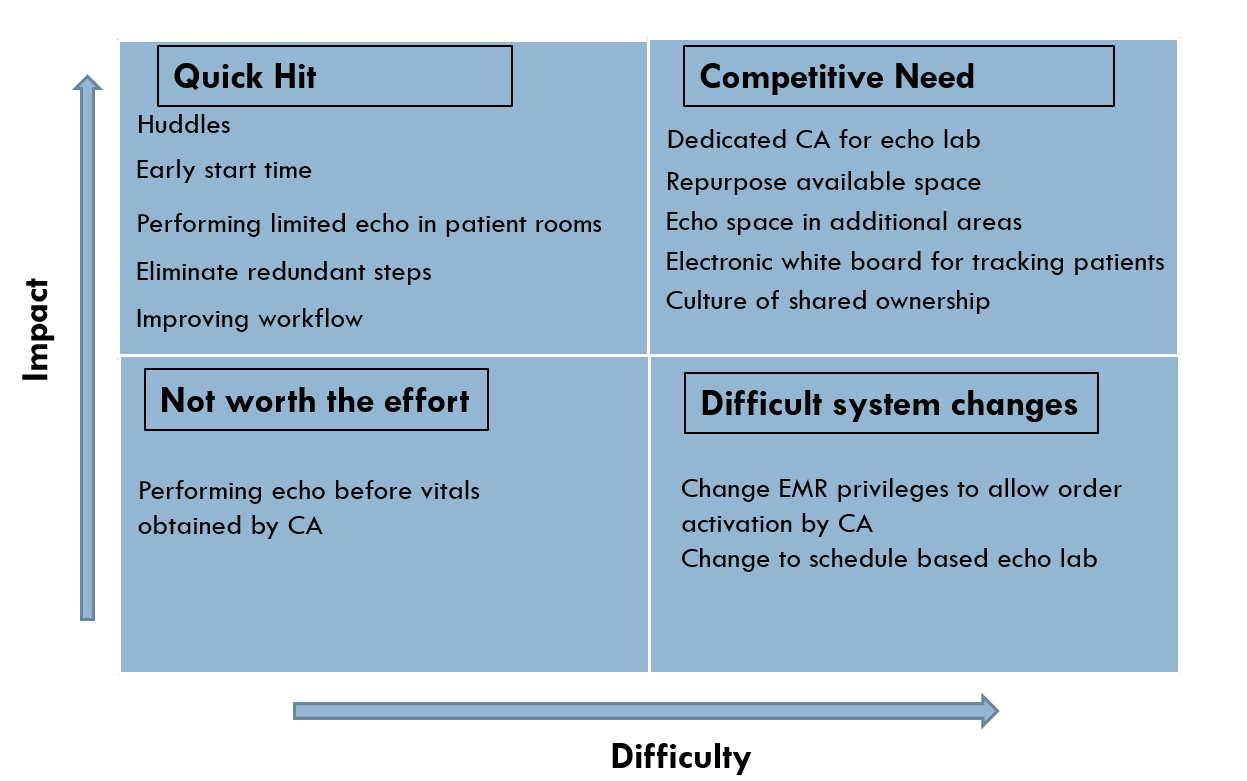

Supplement: Supplementary file 1 [file pqs-3-e083-s001.tif]
